# Superconductivity in planarised nanocrystalline diamond films

**DOI:** 10.1080/14686996.2017.1286223

**Published:** 2017-03-23

**Authors:** Georgina M. Klemencic, Soumen Mandal, Jessica M. Werrell, Sean R. Giblin, Oliver A. Williams

**Affiliations:** ^a^ School of Physics and Astronomy, Cardiff University, Cardiff, UK.

**Keywords:** Boron doped diamond, surface roughness, chemical mechanical polishing, chemical vapour deposition, superconductivity, 10 Engineering and Structural materials, 104 Carbon and related materials, 212 Surface and interfaces, 303 Mechanical / Physical processing, 306 Thin film / Coatings, 203 Magnetics / Spintronics / Superconductors

## Abstract

Chemical vapour deposition (CVD) grown boron-doped nanocrystalline diamond (B-NCD) is an attractive material for the fabrication of high frequency superconducting nanoelectromechanical systems (NEMS) due to its high Young’s modulus. The as-grown films have a surface roughness that increases with film thickness due to the columnar growth mechanism. To reduce intrinsic losses in B-NCD NEMS it is crucial to correct for this surface roughness by polishing. In this paper, in contrast to conventional polishing, it is demonstrated that the root-mean-square (RMS) roughness of a 520 nm thick B-NCD film can be reduced by chemical mechanical polishing (CMP) from 44.0 nm to 1.5 nm in 14 hours without damaging the sample or introducing significant changes to the superconducting transition temperature, $T_{C}$, thus enabling the use of B-NCD films in the fabrication of high quality superconducting NEMS.

## Graphical Abstract

A CVD-grown boron-doped diamond film was polished to a surface roughness of <2 nm RMS. The superconducting properties of the film are unchanged by the polishing process.

**Figure:**
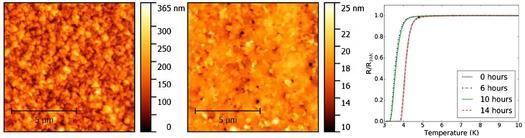

## 1. Introduction

Diamond is a material of extreme properties of particular use in the fabrication of nano-electro-mechanical systems (NEMS) for the study of macroscopic quantum objects [1]. The thermal occupation of such a device is defined as $\bar{n}=\left(k_{B}T_{R}/hf_{R}\right)-1/2$, where $f_{R}$ is the resonant frequency and $T_{R}$ is the resonator temperature. The ground state is reached under the condition $\bar{n}\leq 1$; this is achievable at higher temperatures if $f_{R}$ is maximised. The resonant frequency of a doubly clamped beam is proportional to the acoustic velocity of the material; thus, with a Young’s modulus of 1200 GPa [2], diamond is an attractive candidate for use in the fabrication of NEMS with higher $f_{R}$ than are achievable in more conventional materials. Remarkably, diamond retains many desirable mechanical properties when synthesised as a nanocrystalline diamond (NCD) film by chemical vapour deposition (CVD), with the advantages of large area thin film wafer coating that facilitates planar circuit fabrication at a considerably reduced price compared to single-crystal growth [3–5]. The use of silicon wafers also allows for the substrate undercutting that is often required in NEMS fabrication processes. Indeed, NEMS fabrication from NCD films has already been demonstrated with device performances that rival or even surpass those of more conventional materials, but are noted to be limited by the considerable surface roughness that arises from the columnar CVD growth mechanism unless care is taken to correct for this [6–8] or use single-crystal diamond [9,10].

To detect the displacement of a doubly clamped NEMS device, it may be directly incorporated into the loop of a superconducting quantum interference device (SQUID) as successfully demonstrated by Etaki et al. [11]. This introduces another constraint on the material of the NEMS device – that it should be superconducting. Through the introduction of boron as a charge acceptor into the carbon lattice during synthesis, heavily doped diamond also exhibits superconductivity [12] in both single-crystal [13] and NCD films [14]. It is the combination of superconductivity and extrememechanical properties that provides an opportunity to improve on the fabrication of superconducting NEMS to drive forward the field of quantum nanomechanics. Successful fabrication of superconducting diamond devices [15], including NEMS [16], has already been demonstrated with unpolished boron doped nanocrystalline diamond (B-NCD), though it was again noted that smoothing the surface would improve device performance.

If B-NCD is to be used to fabricate high quality devices, the problematic as-grown surface roughness must be corrected [6,8]. Despite the extreme hardness of diamond, there are a number of polishing methods that can be implemented to correct the as-grown surface roughness [17,18], though it is well known that some can result in subsurface sample damage that could potentially degrade the superconducting properties [19,20]. For example, Wu et al. [21] reported an unexpected loss of superconductivity after mechanical polishing. From the described experiment, it is not clear if the sample no longer showed superconductivity, or if $T_{C}$ was lowered to a temperature that was simply not achievable with their experimental apparatus. While X-ray diffraction (XRD) revealed no significant structural changes induced by the polishing process, the boron concentration was reported to be reduced by approximately six times the original value. Though this may suggest an inhomogeneous boron distribution in the sample, the overall conclusion from their work should be that mechanical polishing of B-NCD films can have marked effects on the superconducting properties.

An alternative to the mechanical polishing of NCD films is chemical mechanical polishing (CMP), a technique commonly used in industrial integrated circuit fabrication processes. This technique replaces the cast iron scaife with a soft polyester-based pad and has recently been demonstrated to be effective in polishing both single-crystal diamond and NCD films [22,23]. While mechanical polishing of diamond is highly anisotropic [24], which limits material removal rates, CMP removal rates have been shown to be ∼16 nm $h^{-1}$ in NCD films [23]. This rate is comparable with that obtained by mechanical polishing [18] but the technique has the advantage of using significantly lower forces which prevent shattering of thin film samples that are stressed or have significant bow across the wafer. Furthermore, and more importantly for this work, a study of CMP on single-crystal diamond showed that there is no evidence of fracture damage whilst polishing along hard directions, and the lack of diamond grit as an abrasive leads to the assumption of little to no subsurface damage to the sample [22]. In this paper, we demonstrate the successful application of CMP on a B-NCD film without inducing significant changes to the as-grown superconducting transition temperature. The immediate application of this polishing technique is for use on B-NCD films intended for superconducting NEMS fabrication.

## 2. Experimental methods

### 2.1. B-NCD film growth

The B-NCD film used for this study was grown using microwave plasma-assisted CVD [25]. The substrate was an SC-1 cleaned 2" diameter, 500 μm thick (100) silicon wafer with a 500 nm thick $\;{\text{SiO}}_{2}$ buffer layer. Prior to film growth, the substrate was seeded by ultrasonic agitation for 10 min in a monodisperse aqueous colloid of nanodiamond particles. This method is known to produce uniformly spaced nucleation sites with a density in excess of $10^{11}$ ${\mathrm{cm}}^{-2}$, thereby reducing the initial as-grown surface roughness and producing high quality fully coalesced thin films with uniform grain sizes across the substrate [26]. The substrate was then rinsed in pure deionised water, spun dry, and loaded directly into the reactor chamber. The film was grown in a dilute gas mixture of methane and trimethylboron in hydrogen, with a 3% methane concentration and a B/C ratio of 12,800 ppm, using a Seki AX6500 series microwave plasma reactor supplied by Seki Technotron, now Cornes Technologies Limited, Japan. The chamber pressure and microwave power were 40 Torr and 3.5 kW respectively. The substrate temperature was ∼720${}^{\circ }$C during growth, as measured in situ with a dual wavelength pyrometer. The as-grown film thickness was determined by *in situ* pyrometric interferometry to be 520 nm. This thickness was obtained after a deposition time of 150 min, after which the film was cooled in a hydrogen plasma and subsequently removed from the reactor chamber.

### 2.2. Transition temperature measurement

The resistive superconducting transition of the B-NCD film was measured before and after CMP processing. Whole wafers are required for the polishing sample holder, therefore the complete 2${}^{\prime \prime }$ wafer was clamped to a variable temperature stage on the cold plate of a pumped liquid ${}^{4}$He cryostat. A diode thermometer and a surface mount heater were glued centrally to the exposed underside of the wafer. A symmetrical jig with a 1 cm${}^{2}$ arrangement of spring loaded pins was pushed directly onto the film surface and a four-terminal measurement of the film resistance was made using an AVS-47B AC resistance bridge. To measure $T_{C}$, the heater power was ramped at a rate of 5.56 μWs${}^{-1}$, and the diode thermometer was monitored. Following the low-temperature measurement of the unpolished film, the sample was removed from the cryostat, the thermometer and heater were removed, and the wafer was incrementally polished as described below.

### 2.3. Film polishing

The B-NCD film was polished by CMP using a Logitech Tribo polishing system equipped with a polyurethane impregnated polyester felt (Suba X, Engis (USA)) polishing pad with Logitech SF-1 Polishing Fluid – an alkaline colloidal silica polishing slurry. The polishing technique and proposed mechanisms are well described elsewhere [22,23,27]. Prior to polishing, the felt pad was roughened with a chuck embedded with diamond grit for 30 min to promote the surface texture optimally required for polishing [28]. In contrast with other polishing techniques, the diamond grit remains in the conditioning chuck and does not make contact with the wafer to be polished. Following pad conditioning, the sample and the polishing pad were rotated in opposite directions at 60 rpm with a down-load of 2 psi (13.8 kPa), a slurry feed rate of 40 ml min${}^{-1}$. A coarse attempt to correct for the wafer bow (typically ∼4 μm across a wafer) was made by pneumatically applying 20 psi (138 kPa) of back pressure to the wafer. During polishing, *in situ* conditioning was used throughout to maintain the optimal surface texture of the polishing pad. The film was polished intermittently for 14 h in total, with $T_{C}$ measurements made as the surface roughness decreased. Following each polishing step, the film was SC-1 cleaned for 20 min to remove remaining slurry from the surface. The surface morphology at decreasing surface roughness was studied by atomic force microscopy (AFM) using a Park Systems XE-100 AFM, and scanning electron microscopy (SEM) using a Raith eLine system.

**Figure 1.**
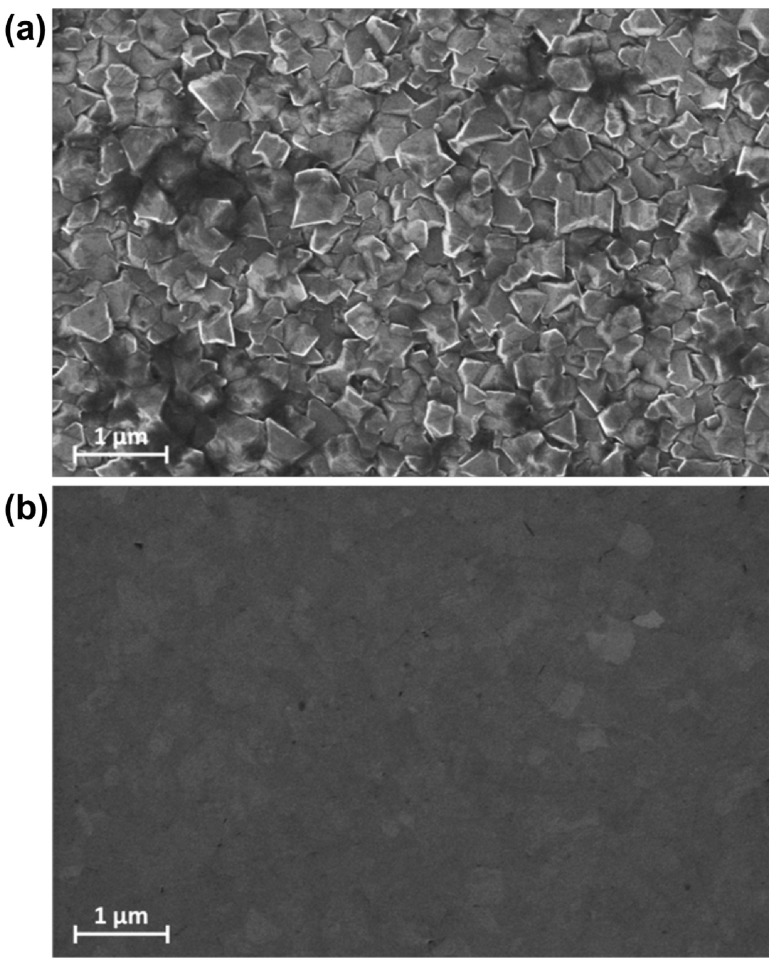
SEM images of the (a) as-grown and (b) 14-hour polished film. The as-grown film had clearly defined grains of average size ∼280 nm. The surface roughness was 44.0 nm RMS in (a) and 1.5 nm RMS in (b).

## 3. Results and discussions

### 3.1. Surface morphology

Figure 1 shows SEM images of the as-grown (a) and polished (b) B-NCD film. The micrograph (a) shows a fully coalesced film with an average grain size of ∼280 nm. The polycrystalline nature of the film is apparent, with no single predominate growth direction emerging at this thickness and the surface roughness of the as-grown film is evident, arising from the random crystal orientations and columnar crystal growth [25]. After polishing for 14 h, Figure 1(b) shows the surface of the same film. The gentle polishing mechanism of CMP is clearly shown – the surface roughness is reduced as the randomly orientated crystal peaks in contact with the polishing pad are removed concurrently. This action continues at pace until neighbouring crystals are the same height. Since the material removal mechanism is chemo-mechanical in nature [23,27], there is no evidence of the fracture damage commonly seen when mechanical abrasion techniques are applied to CVD-grown diamond [18].

**Figure 2.**
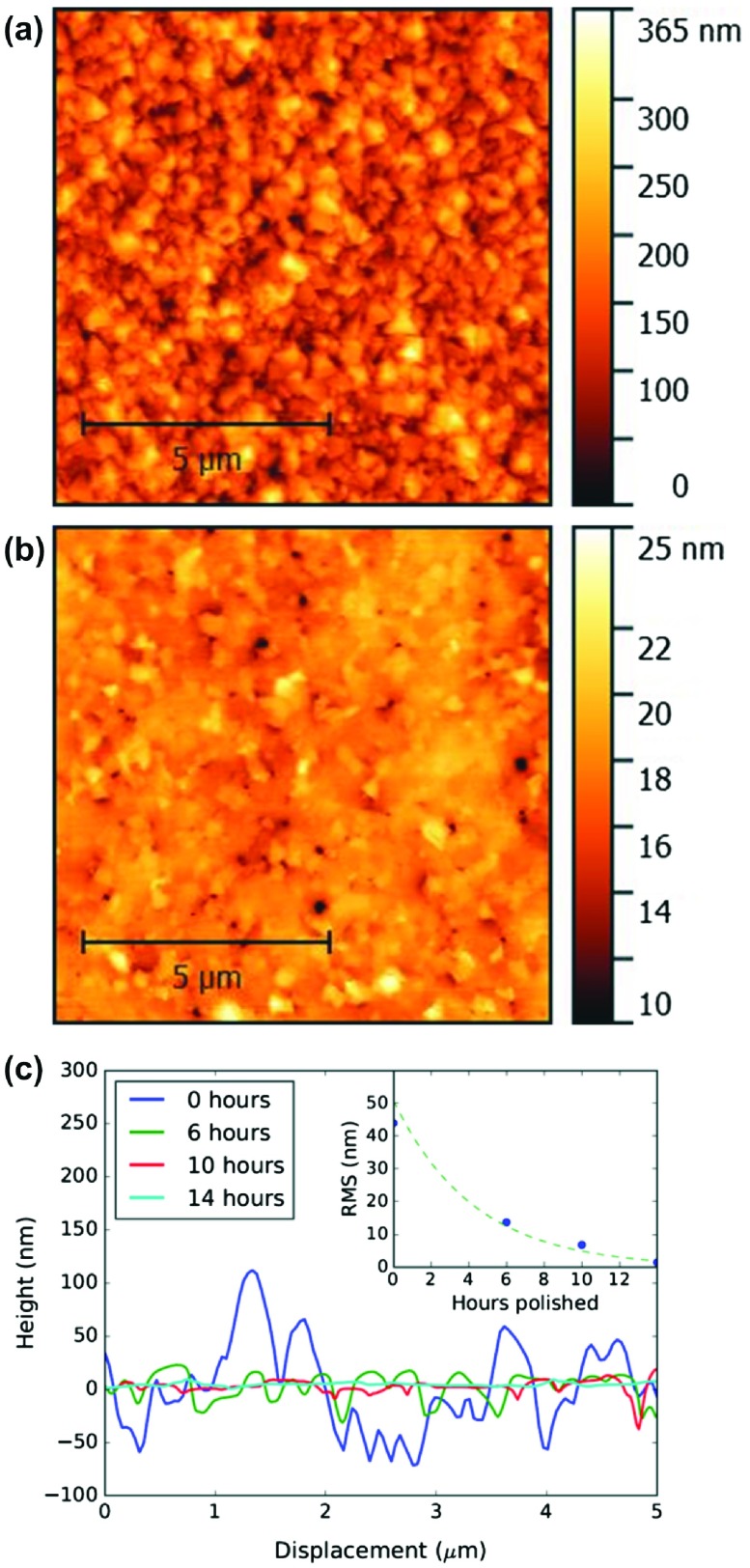
AFM images for the (a) as grown and (b) 14-h polished film. Line traces across the centre of each AFM micrograph are shown in (c) to illustrate the smoothing of the film surface by CMP over time. Inset: the RMS surface roughness as the film is polished.

Corresponding AFM micrographs are shown in Figure 2 for the as-grown (a) and polished (b) film over an area of 100 μm${}^{2}$ located in the centre of the B-NCD film. Figure 2(c) shows lines traces across the centre of AFM images for the unpolished and polished film, as well as at various stages of polishing, as determined using Gwyddion SPM analysis software [29]. The inset shows the RMS surface roughness for increasing polishing times starting at 44.0 nm RMS for the unpolished film to a final value of 1.5 nm RMS after 14 h of CMP polishing. These data support the conclusions drawn from the SEM images – that CMP is a gentle polishing method that does not introduce damage to the remaining B-NCD film material. The images show an absence of debris or residue on the surface, confirming that the post-polish SC-1 clean is sufficient to remove any material and slurry from the CMP process and, further to this, Thomas et al. [23] showed that the surface chemistry is largely unchanged by the CMP process. Importantly, the SEM and AFM micrographs do not show evidence of fracture damage to the B-NCD film as can be observed when polishing with other techniques [17].

**Figure 3.**
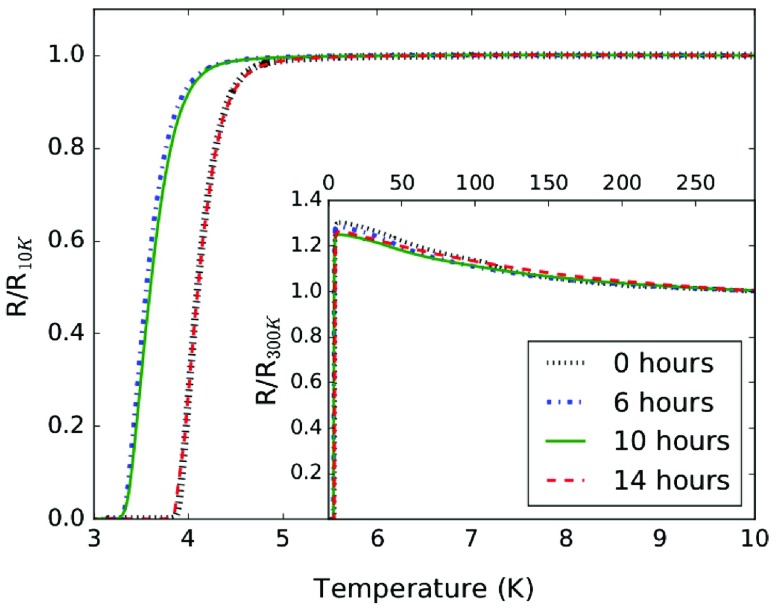
Resistive superconducting transition of the B-NCD film at various stages of polishing. The inset shows the variation of resistance with temperature from 1.6 K to room temperature. The measurement of *R*(*T*) for the 14-h polished film is dashed (red) to show that it lies almost exactly on top of the data for the as-grown film (black).

### 3.2. Superconducting transition temperature

To assess the extent to which CMP affects the superconducting transition temperature, the variation of the normalised resistance with temperature is shown in Figure 3 for the B-NCD film at varying stages of polishing. The inset shows the variation of normalised resistance with temperature from 1.6 K to room temperature. There is an overall increase in the measured sample resistance as the temperature is lowered from 300 K to the onset of superconductivity. This has been observed in freestanding films [12,30], though here the film was grown on a p-doped silicon substrate with a quoted room temperature resistivity of 1–20 Ωcm which makes it difficult to disentangle the behaviour of the B-NCD material and the substrate itself over this temperature range. Focusing on the temperature range below 10 K, the as-grown film had a $T_{C}$ = 4.2 K with a transition width, $\Delta T_{C}$ = 0.5 K. Following the 14 h of CMP processing needed to reduce the surface roughness to <2 nm RMS, the values of $T_{C}$ and $\Delta T_{C}$ are unchanged. In Figure 3, these data are dashed to clearly show that they lie on top of those for the unpolished film. The most important overall result here is that the B-NCD film remains superconducting without a considerable change to $T_{C}$ after over 10 h of polishing. The persistence of superconductivity has been observed for a number of CMP-processed B-NCD films. This result clearly demonstrates that, in contrast to the findings of Wu et al. [21] which suggests that mechanical polishing can significantly change or even destroy the superconductivity in B-NCD films, CMP does not destroy the superconductivity as measured by the resistive transition temperature.

Following the first six hours of polishing, $T_{C}$ is initially reduced by 0.5 K with a corresponding increase in the width of the transition by 0.1 K. The measured resistive $T_{C}$ then increases until the original value is recovered, though the reason for this is not immediately clear. An important experimental detail here is that the measurement is of the resistive $T_{C}$ and is made by pressing contacts directly onto the surface of an unpatterned 2${}^{\prime \prime }$ (51 mm) wafer. The I–V characteristics of the contacts were checked for linearity at several temperatures, but it could not be guaranteed that the contacts were made to identical points on the wafer for each $T_{C}$ measurement. If there is a local variation in $T_{C}$, this could result in small measurable differences. Additionally, it is possible that as material is removed from the surface, some percolative pathways through the film are removed which could lead to a broadening of the transition width. As the film is polished, a reduction in the average grain size could also lead to changes in $T_{C}$ and $\Delta T_{C}$ [31]. Since CMP is a gentle method of polishing involving small mechanical forces, it is not believed that there is any subsurface damage to the film in the same way it is introduced in mechanical polishing. In contrast to the findings of Wu et al. [21] that suggest that mechanical polishing can significantly change or even destroy the superconductivity in B-NCD films, this work finds that CMP does not destroy the superconductivity as measured by the resistive transition temperature.

## 4. Conclusions

To conclude, it has been shown that a B-NCD film of as-grown surface roughness 44.0 nm RMS retains superconductivity following more than 10 h of chemical mechanical polishing, until a surface roughness of 1.5 nm RMS is reached. The observed $T_{C}$ was 4.2 K for the unpolished film and, after an initial reduction of 0.5 K, remained superconducting thereafter until the original value was recovered. The importance of this result is that superconductivity is not destroyed following the use of CMP, thus thin CVD-grown boron doped diamond films can be polished to a smooth surface finish of <2 nm RMS and used to fabricate superconducting NEMS devices. Following this process, energy loss mechanisms in NEMS arising from surface roughness will be reduced, and therefore this work finds immediate application in the field of quantum nanomechanical devices.
